# Enhancing intraoral radiographic technique: introducing “Nallan's Lines”

**DOI:** 10.3389/froh.2024.1498117

**Published:** 2024-12-20

**Authors:** Nallan CSK Chaitanya, Vivek Padmanabhan, Md Sofiqul Islam, Nada Tawfig Hashim, Riham Mohammed, Neeharika Satyajyothi Allam, Jouma Jalal Omar, Ahmed Zakaria, Shaga Pranathi, Mamindla Apoorva

**Affiliations:** ^1^ Department of Oral Radiology and Oral Medicine, RAK College of Dental Sciences, RAK Medical and Health Sciences University, Ras Al Khaimah, United Arab Emirates; ^2^ Department of Pedodontics, RAK College of Dental Sciences, RAK Medical and Health Sciences University, Ras Al Khaimah, United Arab Emirates; ^3^ Department of Restorative Dentistry, RAK College of Dental Sciences, RAK Medical and Health Sciences University, Ras Al Khaimah, United Arab Emirates; ^4^ Department of Periodontics, RAK College of Dental Sciences, RAK Medical and Health Sciences University, Ras Al Khaimah, United Arab Emirates; ^5^ Department of Oral Surgery, RAK College of Dental Sciences, RAK Medical and Health Sciences University, Ras Al Khaimah, United Arab Emirates; ^6^ Department of Dentistry, Tatva Dental Center, Hyderabad, India; ^7^ RAK College of Dental Sciences, RAK Medical and Health Sciences University, Ras Al Khaimah, United Arab Emirates; ^8^ Department of MS healthcare Informatics, Sacred Heart University, Fairfield, CT, United States; ^9^ Department of Oral Medicine and Radiology, Panineeya Dental College, Hyderabad, India

**Keywords:** intraoral radiography, geometric distortion, periapical projections, gyroscope, radiographic reference points

## Abstract

**Background:**

Intraoral radiography remains the most widely employed dental radiographic technique for producing geometrically accurate images with minimal distortion and magnification. Despite its common use in the dental office, incorrect projection geometry can be challenging leading to image distortions. To mitigate these inaccuracies specific radiographic landmarks on the face are utilized during periapical radiography. Nallan's lines, proposed in this study may address the perpetual issue.

**Material and methods:**

In this cross over study, the participants were divided into 2 groups, Group A and Group B comprising nine in the each group. The bisecting angle technique was performed by the participants using a gyroscopic device fitted with a LASER with (Study subjects) and without (Controls) utilizing Nallan's lines on a selected group of teeth using a phantom model. Subsequent images were compared with those images obtained by an oral radiologist using the same model for geometric distortions of the images. This comparative analysis aimed to identify potential geometric distortions within the generated images

**Results:**

A paired *t*-test was employed to compare the mean length and breadth of teeth before and after radiographic training. Additionally, a McNemar Test was used to assess the impact of Nallan's lines on technical errors. Both analyses revealed statistically significant improvements post-training (*p* < 0.01). To determine if radiographic training affected the performance of volunteers and specialists, a Chi-Square Test was conducted to compare error rates. No statistically significant differences were observed between the two groups, both before and after training.

**Conclusion:**

The adoption of Nallan's lines may enhance the accuracy and quality of intraoral radiographic images. By adhering to this geometric framework dental practitioners can minimize geometric distortions and thereby repeated radiographic exposures of patients.

## Introduction

Intraoral radiography, utilizing the bisecting technique, is a fundamental diagnostic approach in dentistry with its precession relying significantly on accurate film positioning and the careful angulation of x-ray beam (1–3). A dental radiologist primary responsibility during this procedure is to ensure optimum film placement through meticulous assessment of both the horizontal and vertical angulation of the x-ray beam (2). Errors in angulation may lead to image distortions such as elongation or foreshortening of images (2–4). These technical inaccuracies not only reduce the diagnostic quality of radiographic images but can also result in misdiagnosis and treatment failures, while potentially increasing radiation exposure to patients (1–4). To address these issues radiographic landmarks are utilized to improve positioning accuracy and minimize the need for repeated exposure (2).

**Figure 1:**
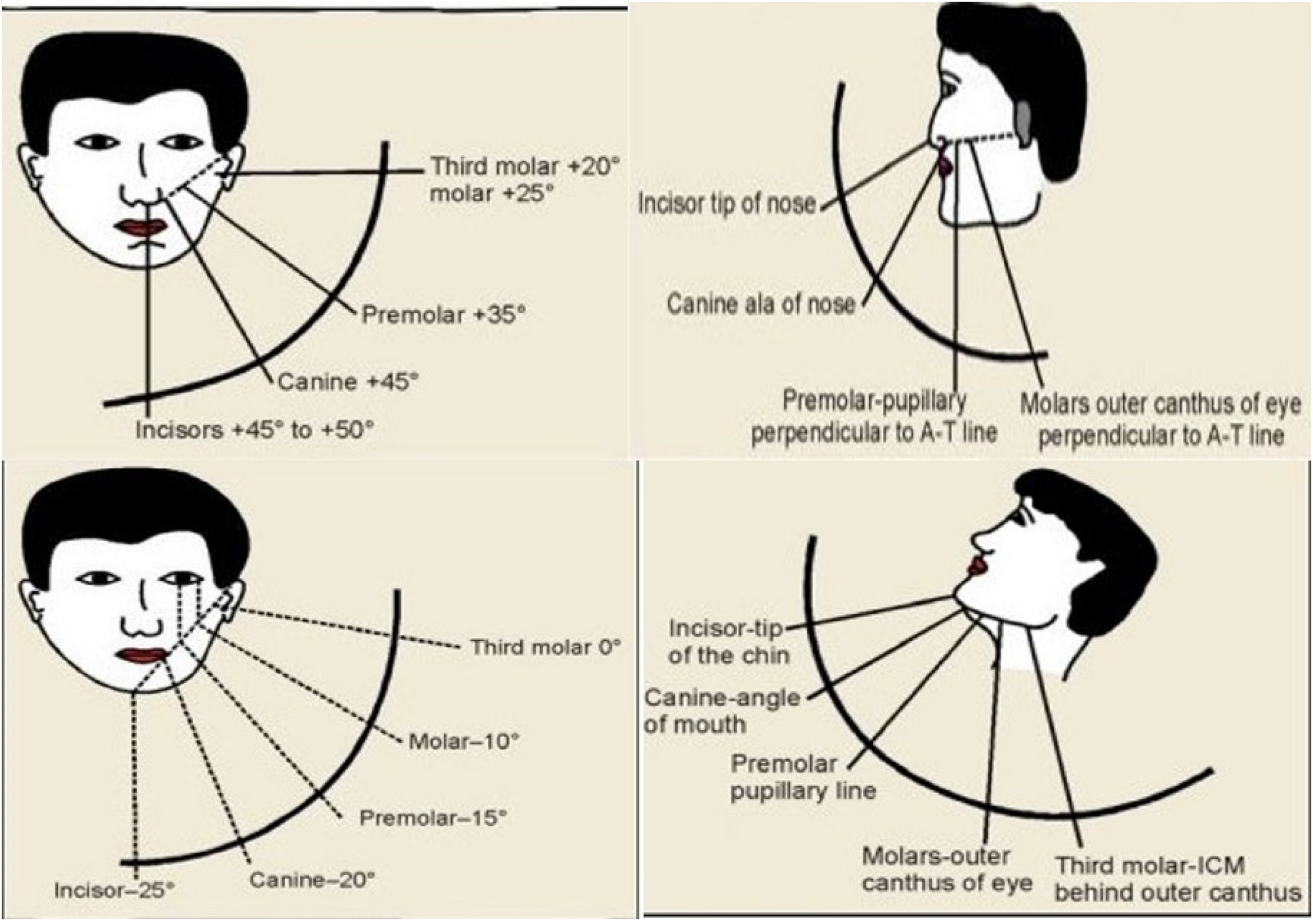
Standard conventional radiographic reference points for maxillary and mandibular teeth periapical projections.

Standard reference points for intraoral radiography are well-established, with the Ala-tragus line being a crucial guide for directing the x-ray beam in both maxillary and inferior border of mandible for mandibular radiographic exposures. Additionally various anatomical landmarks in the maxilla and mandible serve as standard reference points for x-ray beam alignment particularly in the bisecting technique (1–4) (Figure 1).

However the event effectiveness of these landmarks may be limited by anatomical variations and overlapping structures. Studies have shown that using the ala-tragus line as an anatomical reference line in periapical radiographic projections (2) can significantly improve accuracy in periapical imaging compared to those where it was not utilized as a reference. Nonetheless, visualizing the imaginary ala-tragus line presented certain challenges: Clinicians may occasionally rely on the inferior or superior border of the tragus, experience confusion over the posterior reference points, and required substantial skill and training to orient the x-ray beam accurately to the area of interest. Frankfort's horizontal plane could also be used for maxillary teeth radiographic positions. Furthermore, barring the usage of inferior border of mandible, as a guidance plane, there are no specific radiographic reference lines available for mandibular teeth.

In response to these limitations, a pilot study was conducted to evaluate the effectiveness of modified radiographic reference guidance termed “Nallan's Lines.” This study aimd to offer a more precise and reliable approach for image acquisition in the bisecting the angle technique. It compared the usage of the standard reference guides and the “Nallan Lines” for maxillary and mandibular intraoral radiographic projections.

### Concept of Nallan's lines

**Figure 2:**
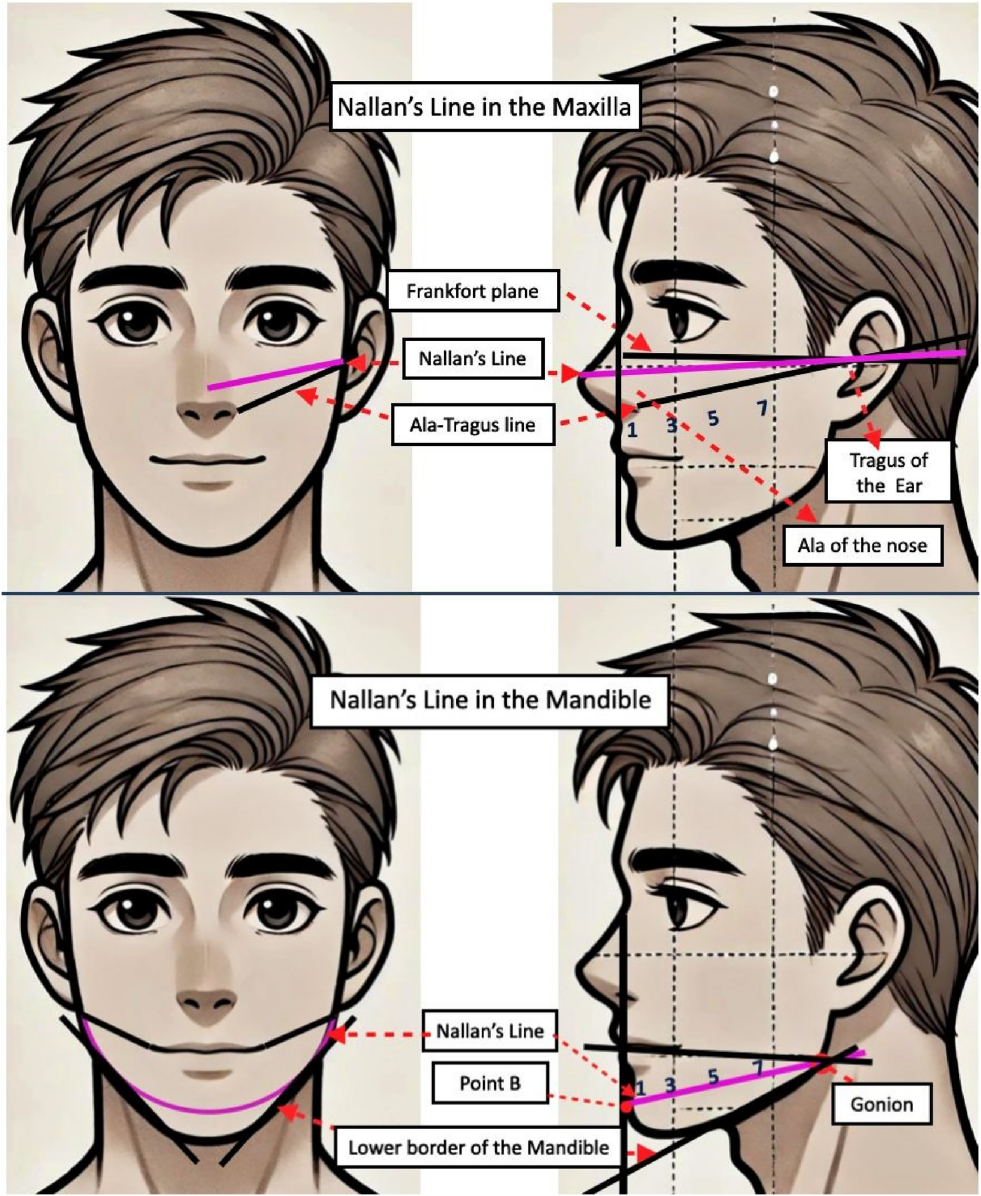
Shows Nallan's line for maxillary and mandibular radiographic projections. (1) Incisors. (3) Canine. (5) Premolars. (7) Molars.

Nallan's lines or Modified radiographic reference lines are confined by specific anatomical boundaries within the mandible and maxilla. In the maxilla, the reference line is an imaginary horizontal line bisecting the triangular region defined by the Frankfort horizontal line, the superior border of the tragus of the ear and the ala of the nose. In the mandible, the line is a horizontal line drawn from there soft tissue of Point B to the gonion on the same side as identify it in this cephalometric analysis. It further bisects the line drawn from corner of mouth to angle of the mandible superiorly and inferior border of the mandible inferiorly (Figure 2).

## Materials and methods

### Study design

A single-centre, prospective, cross-over study was conducted over a 5 month period a t the Radiology Lab of RAKCODS after obtaining necessary ethical approval. The study was designed as an *in vitro* preclinical investigation which involved 9 participants, including general dental practitioners and dental nurses who routinely make radiographic projections in the dental clinics independently. Undergraduate dental students were trained for dental radiology projections in the radiology lab only from the third year of dental course based on the course curriculum. This necessitated more training before they could make intraoral radiographic projections unassisted. Thus they were excluded from participation in the study. Each participant performed two rounds of radiographic procedures within a 30-day period. This allowed for proper training of the personal and no overlap of both tt old and the new projection techniques. Each participant made at least 10 radiographic projections using the bisecting angle technique on the phantom model with and without a LASER assisted gyroscopic device. The projections included selected maxillary and mandibular teeth within the anterior and posteriors. A phantom head equipped with upper and lower jaws was utilized to simulate the human oral cavity for the radiographic procedures. This provided a consistent and reproducible model for all participants, ensuring standardization across the study. Utilizing the Raosoft sample size calculator, the calculated sample size with a 5% margin of error and 95% confidence level is 90.

The participants were divided into two groups: Group A (conventional standard projections) and Group B (Nallan Lines). Group A performed intraoral digital radiography using a CCD sensor and Nallan DirectRay device, following the standard radiographic reference points like the ala-tragus line and the inferior border of mandible. Subsequently the same participants assigned as Group B were trained to use Nallan's Lines as radiographic reference and repeated the procedure. The results of both groups were compared to radiographic procedures performed by a specialist oral radiologist using gyroscope-enabled devices with and without usage of the Nallan's lines. The horizontal and vertical angulations for each of the teeth were followed as standard angulations for both the technique without any change.

**Figure 3:**
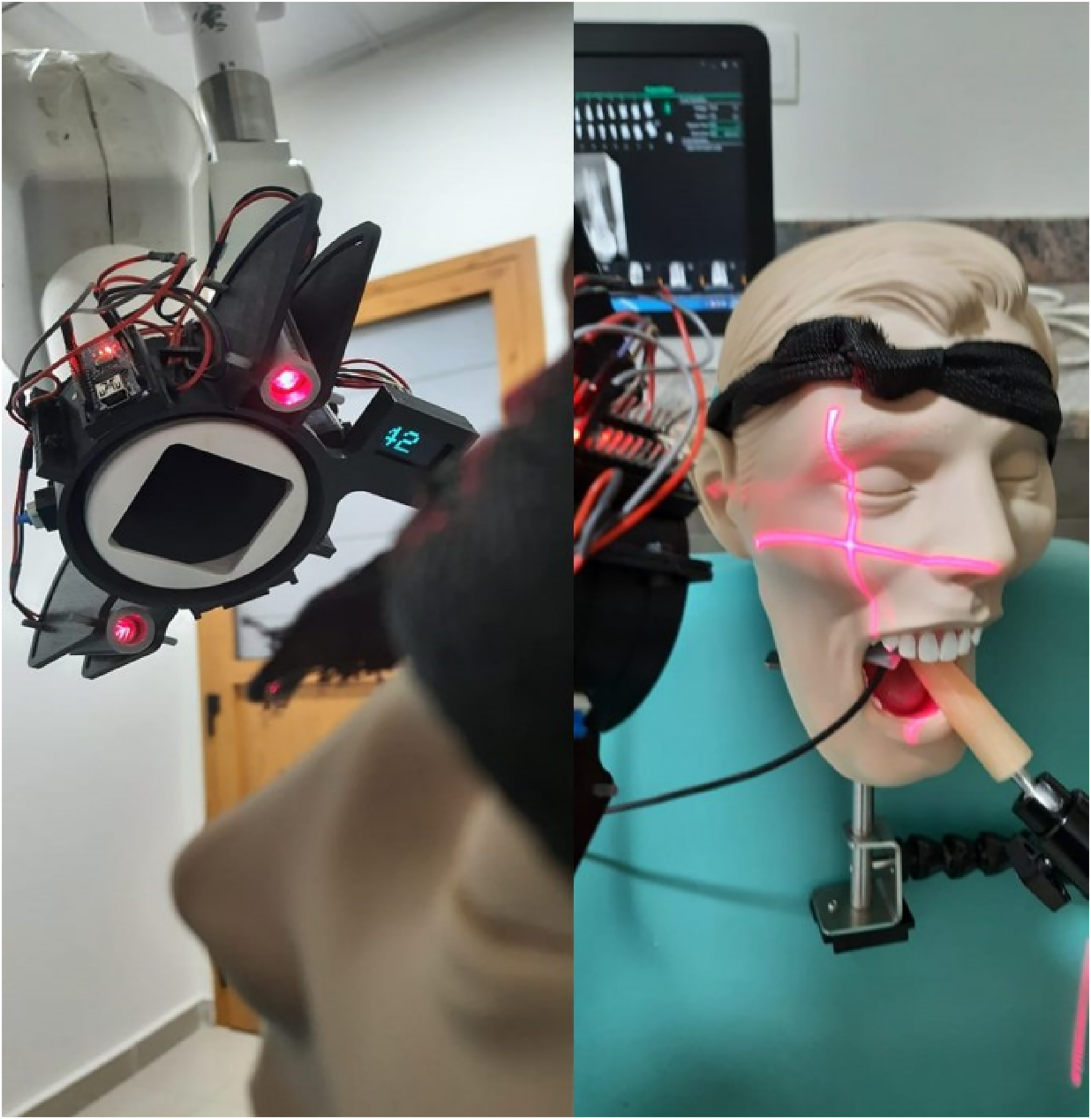
Shows Nallan's directRay LASER and gyroscopic device fitted on the x-ray machine for periapical projections.

Nallan-DirectRay is a gyroscope enabled laser device mounted on the position indicating device of the dental radiographic machine. This was innovatively developed device for precise and accurate radiographic reference points on the area of interest. The validity of the device was checked and is under patent evaluation (Figure 3).

The following equipment was employed for the study:

• **Intraoral x-ray Machine:** Used for generating the radiographic images.
• **CCD Sensor:** A charge-coupled device sensor was used to capture the x-ray images.
• **Nallan DirectRay Device:** Utilized to assist in the accurate positioning of the x-ray beam.
• **Computer:** For storing and analyzing the captured radiographs.
• **Lead Aprons:** Provided to all participants to ensure radiation protection.

The landmarks used as guidance for performing radiographic examinations of maxillary and mandibular teeth with standard and Nallan's Lines are provided in Table 1.

**Table 1:** Radiographic landmarks for maxillary and mandibular teeth with Standard and Nallan's lines (1, 5).

	Standard reference lines	Nallan's lines
Maxillary incisors:	Tip of the nose	1 cm above the tip of the nose, on the bridge of the nose
Maxillary canine:	Ala of nose/Canine eminence	On the Nallan's line above the ala tragus line 1 cm over the canine eminence
Maxillary premolars:	Pupillary perpendicular to A-T line below the pupil of the eye	On the Nallan's line, 1 cm above the ala tragus line with intersection of a perpendicular line below the pupil of the eye.
Maxillary molars:	Outer canthus of the eye perpendicular to ala tragus line./cheek below the outer canthus of the eye and the zygoma at the position of the maxillary second molar	On the Nallan's line, 1 cm above the ala tragus line with intersection of a perpendicular line from outer canthus of eye.
Mandibular incisors:	Tip of the chin below the lower lip and about 1 cm lateral to the midline.	Soft tissue of point B corresponding to the perpendicular line drawn from philtrum of upper lip to inferior border of mandible
Mandibular canine:	Angle of mouth nearly perpendicular to the ala of the nose, over the position of the canine, and about 3 cm above the inferior border of the mandible	Perpendicular line drawn from corner of mouth to inferior border of mandible and a point taken on the horizontal line drawn from soft tissue of point b to gonion (Nallan's line).
Mandibular premolars:	Outer canthus of the eye/below the pupil of the eye and about 3 cm above the inferior border of the mandible.	An imaginary horizontal line drawn from soft tissue of point b to gonion (Nallan's Lines) and perpendicular line drawn from center of the pupil of the corresponding eye to inferior border of the mandible
Mandibular molars:	1 cm away from outer canthus of the eye below the outer canthus of the eye about 3 cm above the inferior border of the mandible.	An imaginary horizontal line drawn from soft tissue of point b to gonion (Nallan's Lines) and a perpendicular line drawn from outer canthus of the corresponding eye to inferior border of the mandible.

The radiographs taken by each participant were saved as digital images and were transferred to a computer. The images were organized and analyzed using Microsoft Excel. Parameters such as image quality, clarity of anatomical landmarks, and technical aspects such as the length and breadth of the teeth projected, foreshortening, elongation, apical cut, cone cut and horizontal overlap were systematically recorded, evaluated and compared between the two groups.

### Statistical analysis

The data were analyzed to evaluate the consistency and accuracy of radiographs taken by both groups. Statistical analysis was performed using SPSS for Windows, Version 16.0 (Chicago, IL). A paired Student's *t*-test was used to compare the mean length and breadth of the tooth (in millimeter) in both the control and study groups before and after training. The McNemar test was employed to compare radiographic errors before and after radiographic training in both groups. level of significance was set at *p* < 0.05. Bonferroni Correction and Fischer exact test were also performed address issues with small sample size. Intra observer variability obtained for oral radiologist images was 0.8 kappa value.

## Results

a. **Images from the Specialist**: A paired *t*-test was used to compare the main length and breadth of teeth (in mm) in radiographs taken by specialist oral radiologist before and radiographic training. No statistically significant differences were found in two length and breadth additionally McNemer test revealed no statistically significant difference in radiographic errors before and after training (Table 2).
b. **Images from the volunteers**: A paired *t*-test was used to compare the main length and breadth of teeth before and after radiographic training in volunteers. A statistically significant difference (*p* < 0.01) was observed in tooth length. The McNemer test revealed significant reduction in elongation, apical cut, and horizontal overlap errors after training. While improvements were noted in foreshortening and cone cut errors, these differences were not statistically significant (Table 2). These findings suggest that training in the use of Nallan's line as a new radiographic reference significantly improved radiographic image accuracy (Table 3).
c. **Comparison of both specialist and volunteer radiographic images**: A chi-square test was used to compare radiographic errors between volunteers and the specialist radiologist before and after training. No statistically significant differences were found between the two groups for any type of radiographic error both pre- and post-training. However despite the lack of statistical significance, observable improvements were noted in the volunteers' performance after training, particularly in reducing the frequency of foreshortening and apex cut errors. These improvements suggest that radiographic training had a positive impact on reducing certain types of radiographic errors among study subjects, bringing their performance closer to the standard set by the specialist radiologist (Tables 4A, 4B).
d. **Bonferroni Correction**: Initially, the *p*-values were calculated for each type of radiographic error (e.g., foreshortening, elongation, apex cut, cone cut, and horizontal overlap) to compare the study subjects with the gold standard (radiologist). All parameters (Foreshortening, Elongation, Apex Cut, Cone Cut, and Horizontal Overlap) showed non-significant differences in radiographic error rates between study subjects and the radiologist, both before and after using Nallan's lines. Adjusting the *p*-values (e.g., with the Bonferroni correction) was important because multiple chi-square tests were performed to compare radiographic error rates between study subjects and the gold standard, both before and after intervention. The adjustment ensured that any “significant” findings were genuinely meaningful and not the result of chance due to the multiple comparisons made. After applying the Bonferroni correction to account for multiple comparisons, the adjusted *p*-values increased, and all were effectively rounded up to 1.0. This strict correction method minimized the chance of obtaining a false positive result (Type I error) across the multiple tests conducted. With the Bonferroni correction, no differences reached statistical significance during comparison. There was requirement of extra evidence to conclude that Nallan's lines, by increasing the sample size, made a significant impact on reducing radiographic errors when comparing study subjects to the radiologist's standards. Please refer to Table 3 for detailed *p*-values and adjusted *p*-values (Table 5).
e. **Fisher's Exact Test**: To verify the findings and address potential issues with small sample sizes, Fisher's Exact Test was conducted for each parameter, as this test was more suitable for contingency tables with low frequencies in certain categories. Specifically, the Fisher *p*-values were as follows: for foreshortening, 0.47 (before) and 0.36 (after); for elongation, 0.46 (before) and 1.00 (after); for apex cut, 0.35 (before) and 1.00 (after); for cone cut, 0.55 (before) and 1.00 (after); and for horizontal overlap, 0.94 (before) and 1.00 (after). (Table 6)

**Table 2:** Shows comparison of mean length & breadth of the tooth (in mm) and radiographic errors by specialist radiologist with and without Nallan's lines.

Comparison of mean Length & Breadth of the tooth (in mm) before and after using Nallan's lines by Student Paired *t* test						
Parameters	Time	*N*	Mean	SD	Mean Diff	*p*-value
Length	Before	10	23.35	3.17	−0.39	0.70
	After	10	23.74	4.93		
Breadth	Before	10	8.29	1.81	0.07	0.70
	After	10	8.22	1.74		
Comparison of radiographic errors before and after using Nallan's lines by McNemar Test						
Parameter	Category	Before		After		*p*-value
		*n*	%	*n*	%	
Foreshortening	Absent	6	60%	10	100%	0.13
	Present	4	40%	0	0%	
Elongation	Absent	6	60%	10	100%	0.13
	Present	4	40%	0	0%	
Cone Cut	Absent	10	100%	9	90%	1.00
	Present	0	0%	1	10%	
Apical Cut	Absent	7	70%	9	90%	0.63
	Present	3	30%	1	10%	
Horizontal Overlap	Absent	7	70%	8	80%	1.00
	Present	3	30%	2	20%	

**Table 3:** Shows comparison of mean length & breadth of the tooth (in mm) and radiographic errors by participant groups with and without Nallan's lines.

Comparison of mean Length & Breadth of the tooth (in mm) [study subjects] before and after using Nallan's lines by Student Paired *t* test						
Parameters	Time	*N*	Mean	SD	Mean Diff	*p*-value
Length	Before	81	22.05	4.15	1.29	0.01*
	After	81	20.76	3.75		
Breadth	Before	80	7.77	2.12	0.27	0.28
	After	80	7.50	2.19		
Comparison of radiographic errors [study subjects] before and after using Nallan's lines by McNemar Test						
Parameter	Category	Before		After		*p*-value
		*n*	%	*n*	%	
Foreshortening	Absent	65	72.2%	72	81.8%	0.22
	Present	25	27.8%	16	18.2%	
Elongation	Absent	66	73.3%	81	91.0%	0.003*
	Present	24	26.7%	8	9.0%	
Cone Cut	Absent	76	84.4%	72	80.0%	0.50
	Present	14	15.6%	18	20.0%	
Apical Cut	Absent	49	54.4%	82	91.1%	<0.001*
	Present	41	45.6%	8	8.9%	
Horizontal Overlap	Absent	57	63.3%	75	83.3%	0.001*
	Present	33	36.7%	15	16.7%	

**Table 4A:** Comparison of mean length & breadth of the tooth (in mm) b/w study subjects and radiologist before and after using Nallan's lines by independent student *t* test.

Parameter	Time	Time	Mean	SD	Mean Diff	*p*-value
Length	Before	Study Subjects	22.08	4.14	−1.27	0.35
		Gold Std.	23.35	3.17		
	After	Study Subjects	20.67	3.78	−3.08	0.02*
		Gold Std.	23.74	4.93		
Breadth	Before	Study Subjects	7.73	2.18	−0.56	0.44
		Gold Std.	8.29	1.81		
	After	Study Subjects	7.55	2.24	−0.67	0.36
		Gold Std.	8.22	1.74		

**Table 4B:** Comparison of radiographic errors between study subjects and radiologist before and after using Nallan's lines by Chi square test.

Parameter	Condition	Study subjects (%)	Gold Standard (%)	*p*-value
Foreshortening	Before Nallan's Lines	27.8	40.0	0.42
	After Nallan's Lines	18.2	0.0	0.14
Elongation	Before Nallan's Lines	26.7	40.0	0.37
	After Nallan's Lines	9.0	0.0	0.32
Apex cut	Before Nallan's Lines	15.6	0.0	0.18
	After Nallan's Lines	20.0	10.0	0.44
Cone cut	Before Nallan's Lines	45.6	30.0	0.35
	After Nallan's Lines	8.9	10.0	0.91
Horizontal overlap	Before Nallan's Lines	36.7	30.0	0.68
	After Nallan's Lines	16.7%	20.0%	0.79

**Table 5:** Chi-Square test results with adjusted *p*-values (Bonferroni correction).

Comparison	Chi-Square *p*-value	Adjusted Chi-Square *p*-value
Foreshortening before	0.42	1.0
Foreshortening after	0.14	1.0
Elongation before	0.37	1.0
Elongation after	0.32	1.0
Apex cut before	0.18	1.0
Apex cut after	0.44	1.0
Cone cut before	0.35	1.0
Cone cut after	0.91	1.0
Horizontal overlap before	0.68	1.0
Horizontal overlap after	0.79	1.0

**Table 6:** Fisher's exact test results with adjusted *p*-values (Bonferroni correction).

Comparison	Fisher *p*-value	Adjusted Fisher *p*-value
Foreshortening before	0.47	1.0
Foreshortening after	0.36	1.0
Elongation before	0.46	1.0
Elongation after	1.00	1.0
Apex cut before	0.35	1.0
Apex cut After	1.00	1.0
Cone cut before	0.55	1.0
Cone cut after	1.00	1.0
Horizontal overlap before	0.94	1.0
Horizontal overlap after	1.00	1.0

## Discussion

This pilot study aimed to evaluate effectiveness of modified radiographic reference lines, termed “Nallan's lines”, compared to conventional standard lines (ala-tragus lines, Frankfurt horizontal plane) in intraoral periapical radiography. The study findings indicated that the implementation of Nallan's lines significantly improved the accuracy of radiographic images, particularly by reducing elongation of images, apical cut and horizontal overlap errors. This study aligned with Al-Safi who suggested developing a new method in the bisecting angle technique to enhance radiographic outcomes rather than relying on the traditional approaches. However, any specific new method or approach for radiographic projections were not discussed (2). The Nallan's lines could represent an evolution in this approach, offering a practical solution for dental practitioners aiming to improve radiographic precision and reduce errors.

The findings demonstrated a statistically significant improvement in the mean length measurements of teeth following radiographic training with Nallan's lines (*p* < 0.01). This indicates that the modified reference lines enhance image positioning accuracy, contributing to more precise diagnosis and treatment planning based on radiographic images. The absence of significant changes in the control group emphasizes the limitations of traditional reference points which might be more susceptible to variations in anatomy and overlapping structures.

Our results also showed substantial reductions in specific radiographic errors in the study group post-training. Notably, elongation errors decreased from 26.7% to 9.0% (*p* < 0.003), and apical cut errors dropped from 45.6% to 8.9% (*p* < 0.001). Horizontal overlap errors also significantly reduced (*p* < 0.001). These improvements underscore the potential of Nallan's Lines to minimize common technical errors, thereby enhancing image quality and reducing the need for repeated exposures, which ultimately supports better patient safety by lowering cumulative radiation doses.

A systematic review highlighted that the reject rates and error sources in dento-maxillofacial radiography, focusing on intraoral, extra-oral, and CBCT imaging. It also projected that rejection rates were highest for intraoral images, especially periapical radiographs (16.38%), primarily due to positioning errors and patient discomfort. This resonated with our work on Nallan's Lines, as improved positioning methods could reduce these issue significantly (6). In the case of extraoral radiography, the reject rate averaged nearly about 4% for panoramic images and 6% for lateral cephalograms, with patient movement as a main error source. The study also noted a 2.77% rejection rate for CBCT images, where patient stability and head position significantly impacted image quality, suggesting that clearer anatomical references and stability tools, similar to the goals of Nallan's Lines, could reduce errors (6). The review also underscored the need for accurate reference points, adequate operator training, and ergonomic adjustments in positioning, which alignd with our findings on Nallan's Lines' effectiveness in enhancing radiographic accuracy and reducing error rates, such as elongation and apical cut (6).

A study investigated the proficiency of general dental practitioners in Saudi Arabia with intraoral radiographic techniques. It highlighted variations in technique availability and ease of use, suggesting that standardized training could enhance accuracy in radiographic projections and image. It underscored the importance of precise training in radiographic positioning for improved diagnostic outcomes and reduced errors (7).

A more recent review demonstrated that though paralleling technique of dental periapical radiography was considered a better approach for endodontic and periodontal treatment plans, the technique might be a disadvantageous for Asian population group because of space insufficiency in maxillary region. Hence a more practical approach with different radiographic reference points were used in this study which may be employed for any population group (8).

The present study was in agreement with the another study describing the periapical radiographic errors related to apical cut and image contrast (9, 10). Some studies also demonstrated the need for check list or usage of rectangular collimation for dental radiographic projections in order to reduce the errors during the procedures and images obtained. It may be noted that though the check list might not had positive significant correlation between check list use and error occurrence, these new recommendations and modifications may improve the quality of images obtained thereby reducing patient radiation exposure (11, 12). These findings are consistent with another similar study emphasizing the importance of accurate reference points in the intraoral radiography.

The present study used an instrument called Nallan's DirectRay. It is a gyroscope enabled laser device mounted on the position indicating device of the dental radiographic machine. The device helped in precise placement of the position indicating device of the x-ray machine on the phantom model with a digital display of vertical angulations. The validity of the device was checked and under patent evaluation.

Shetty et al. (4) discussed the future potential of laser-guided intraoral radiography for improving accuracy in radiographic procedures. Similarly, Chau et al. (13) and Zamani et al. (14) demonstrated that using laser-guided indicators significantly reduced technical errors in radiography (15). Our study supported these findings by demonstrating that Nallan's lines, used in conjunction with gyroscopic laser guided approach, could serve purpose of enhancing accuracy.

When comparing the study subjects to the specialist radiologist, the study group exhibited a closer approximation of the gold standard in terms of radiographic error rates, post-training. Although not all improvements where statistically significant, the trend suggested a positive impact of the modified reference lines on the radiographic accuracy. Despite the lack of statistically significant differences, several aspects suggested that Nallan's lines offer practical value that may not be fully captured by statistical results alone. Clinically, even modest reductions in radiographic errors—such as foreshortening, elongation, and apex cut could enhance diagnostic clarity and patient outcomes. After implementing Nallan's lines, we observed directional improvements across parameters, with increased rates of error-free images in study subjects, approaching the accuracy achieved by the radiologist. In the clinical context, these incremental improvements could be meaningful, particularly for less-experienced practitioners who may benefit from additional guidance in positioning techniques.

Furthermore, Nallan's lines show significant educational potential as a training aid. Visual references, like those provided by this device, could help students and early-career clinicians develop better spatial awareness and technical accuracy, reinforcing proper positioning habits. This is especially valuable in training environments, where proficiency in radiographic positioning can be challenging to master quickly. While these trends may not appear statistically significant in the current study, repeated use and familiarity with Nallan's lines could, over time, lead to more consistent and accurate positioning, ultimately reducing the likelihood of errors in clinical practice.

### Limitations and recommendations

Future research may yield stronger evidence of the device's efficacy, especially with larger sample sizes or longer study durations. A larger cohort could provide the statistical power needed to detect smaller, yet clinically meaningful, effects that a limited sample size might obscure. Similarly, a randomized control trial assessing the device's impact over time could provide a more comprehensive understanding of its benefits, particularly if it focuses on practical improvements in radiographic accuracy within routine clinical settings. Another potential benefit lies in reducing the need for repeat exposures, which enhances patient safety by limiting unnecessary radiation exposure. This aligns with a patient-centric approach to healthcare, where even minor reductions in radiographic errors have broader implications for patient safety and care quality.

## Conclusion

The introduction of Nallan's lines as modified radiographic reference points demonstrated a substantial improvement in the accuracy of intraoral periapical radiographs, with particular effectiveness in reducing elongation, apical cut, and horizontal overlap errors. This advancement suggested that Nallan's lines may offer a robust framework for improving radiographic precision, leading to more accurate diagnosis and enhanced treatment planning.

## Data Availability

The original contributions presented in the study are included in the article/Supplementary Material, further inquiries can be directed to the corresponding author.

## References

[1] Bailoor DM Nagesh KS. Fundamentals of Oral Medicine and Oral Radiology. New Delhi, India: Jaypee Publisher (2005). p. 276–8.

[2] Al-Safi MA. New approach in Bisecting angle technique. Mustansiria Dent J. (2018) 3(1):1–5. 10.32828/mdj.v3i1.611

[3] Woodsend B Koufoudaki E Lin P McIntyre G El-Angbawi A Aziz A Development of intra-oral automated landmark recognition (ALR) for dental and occlusal outcome measurements. Eur J Orthod. (2022) 44(1):43–50. 10.1093/ejo/cjab012 33950251 PMC8789266

[4] Shetty SR Babu S Varkeyachan E. Laser-guided intraoral radiography: a future focus. Imaging Sci Dent. (2014) 44(3):253–4. 10.5624/isd.2014.44.3.253 25279348 PMC4182362

[5] White SC Pharoah MJ. Oral Radiology: Principles and Interpretation. 7th edn Amsterdam: Elsevier, Health Sciences Division (2014). p. 41–63.

[6] Yeung AWK Wong NSM. Reject rates of radiographic images in dentomaxillofacial radiology: a literature review. Int J Environ Res Public Health. (2021) 18(15):8076. 10.3390/ijerph18158076 34360368 PMC8345626

[7] Elangovan S Mahabob MN Jaishankar S Kumar BS Rajendran D. Faulty radiographs: a cross-sectional analysis among dental college students in Namakkal District, Tamil Nadu, India. J Pharm Bioallied Sci. (2016) 8(1):S116–118. 10.4103/0975-7406.191938 27829760 PMC5074011

[8] Yen M Yeung AWK. The performance of paralleling technique and bisecting angle technique for taking periapical radiographs: a systematic review. Dent J (Basel). (2023) 11(7):155. 10.3390/dj11070155 37504221 PMC10378420

[9] Senior A Winand C Ganatra S Lai H Alsulfyani N Pachêco-Pereira C. Digital intraoral imaging Re-exposure rates of dental students. J Dent Educ. (2018) 82(1):61–8. 10.21815/JDE.018.011 29292327

[10] Siddique SN Anwar MA Zaman H Haider I Ahmad A Umair M Quality assessment of periapical radiographs taken by dental assistants using the recent faculty of general dental practice (FGDP) guidelines. Cureus. (2024) 16(9):e68508. 10.7759/cureus.68508 39364472 PMC11447568

[11] Nenad MW Halupa C Spolarich AE Gurenlian JR. A dental radiography checklist as a tool for quality improvement. J Dent Hyg. (2016) 90(6):386–93. 29118160

[12] Shetty A Almeida FT Ganatra S Senior A Pacheco-Pereira C. Evidence on radiation dose reduction using rectangular collimation: a systematic review. Int Dent J. (2019) 69(2):84–97. 10.1111/idj.12411 29959778 PMC9379043

[13] Chau AC Li TK Wong J. A randomized double blinded study to assess the efficacy of a laser-guided collimator on dental radiography training. Dentomaxillofac Radiol. (2006) 35:200–4. 10.1259/dmfr/84342351 16618855

[14] Zamani NA Hekmatian E Khalili H Sadeghi HS. Effect of using laser guided indicator on the reduction of technical errors in periapical radiographies prepared by dental students. J Isfahan Dent Sch. (2010) 6:173–9.

[15] Stabholz A Zeltser R Sela M Peretz B Moshonov J Ziskind D The use of lasers in dentistry: principles of operation and clinical applications. Compend Contin Educ Dent. (2003) 24:935–49. 14733160

